# Penetrating Thoracoabdominal Trauma With a Cryptic Diaphragmatic Injury in a 23-Year-Old Male

**DOI:** 10.7759/cureus.13102

**Published:** 2021-02-03

**Authors:** Zachary A Koenig, Samuel Schick, Ryan Quigley, Jason Turner

**Affiliations:** 1 Medicine, West Virginia University, Morgantown, USA; 2 Surgery, West Virginia University, Martinsburg, USA

**Keywords:** robotic repair, penetrating trauma, radiography, thoracoabdominal trauma, diaphragmatic hernia, stab wound

## Abstract

Traumatic diaphragmatic injuries are a rare entity and can occur in relation to penetrating thoracic and abdominal trauma. The most common clinical features of diaphragm rupture include chest or abdominal bruising, decreased breath sounds, and signs of bowel obstruction. However, the classic signs and symptoms of diaphragmatic injury are not always present and can be obscured even in the highest resolution imaging. This highlights the importance for maintaining a high index of suspicion to make the diagnosis and properly manage these patients. Here, we present a rare case of a 23-year-old male who experienced a laceration to his left thorax and was later discovered to have concurrent diaphragmatic injury despite an initially noncontributory physical exam and imaging findings. The patient subsequently underwent robotic repair of the injury and reduction of herniated contents.

## Introduction

Traumatic diaphragmatic injury is a rare pathology that is reported in less than 0.5% of all trauma cases, according to the National Trauma Data Bank (NTDB) [1]. Injury to the diaphragm can occur via blunt or penetrating mechanisms, but diaphragm injury secondary to penetrating trauma occurs far more commonly than diaphragm injury secondary to blunt trauma. In contrast to blunt injuries, penetrating injuries cause smaller rents that are essentially equal in length to the impalement. In turn, large penetrating injuries may be acutely clinically obvious, but small penetrating diaphragmatic injuries are more likely to be occult even with the highest resolution imaging methods [2]. Complications from misdiagnosis can be life threatening, and as such, initial assessment begins with comprehension of the anatomy, associated injuries, and pitfalls in diagnostic testing will assist in reaching a more rapid diagnosis.

The diaphragm creates a musculotendinous delineation between the positive-pressure abdominal cavity and the negative-pressure thoracic cavity. The diaphragm rises to the fourth thoracic (T4) dermatome on the right and fifth thoracic dermatome (T5) on the left with maximal expiration, but it descends as low as the eighth thoracic dermatome (T8) with maximal inspiration [3]. Any penetrating injury between these vertebral levels should prompt evaluation for diaphragm rupture. Additionally, one must consider the potential for abdominal involvement of stab wounds if the insult occurs below the fourth intercostal space anteriorly, sixth intercostal space laterally, and the eighth intercostal space posteriorly.

Many key organs and structures traverse through or are in close proximity to the diaphragm because it wraps circumferentially around the abdominal wall. Consequently, it is not surprising that diaphragmatic injuries rarely occur in isolation [4]. Two studies estimated that 75% of patients with diaphragm penetration had two or more concurrent organ injuries [5-6]. These organ injuries are typically congruent with findings on physical examination including poor pulmonary excursion, contusions, ecchymoses, and bowel sounds in the thorax in the case of abdominal organ herniation. Injury to the surrounding organs often mirages the diaphragm rupture because clinicians tend to focus on the overt organ injuries rather than the smaller diaphragm injury. Further complicating the matter is that there can be no symptoms to suggest diaphragmatic injury depending on the size and trajectory of the impalement [7].

The advent of helical CT has provided 84% sensitivity and up to 99% specificity for diagnosing diaphragm rupture in polytraumatized patients, which is superior to previous acute imaging modalities. Numerous pattern recognition signs have been identified to facilitate precise identification of these injuries on imaging [8]. However, traditional contrast and noncontrast CT scans do not consistently reveal diaphragm injuries due to similarities in attenuation levels between adjacent structures [9].

Given these complications, the rate of initially missed diaphragmatic injuries or ruptures in nonoperatively managed patients occur more frequently than desired. A missed diaphragmatic injury has the potential to cause delayed herniation and strangulation of abdominal organs into the thoracic cavity through the unrepaired defect in the diaphragm, owing to its high mortality rate. It can also cause atelectasis, pleural effusions, biliary fistulas, and diaphragm paralysis [10]. The possibility of these complications reiterates the need for maintaining a broad range of differential diagnoses in patients with penetrating trauma even in the absence of findings that would suggest diaphragm injury.

## Case presentation

**Figure 1:**
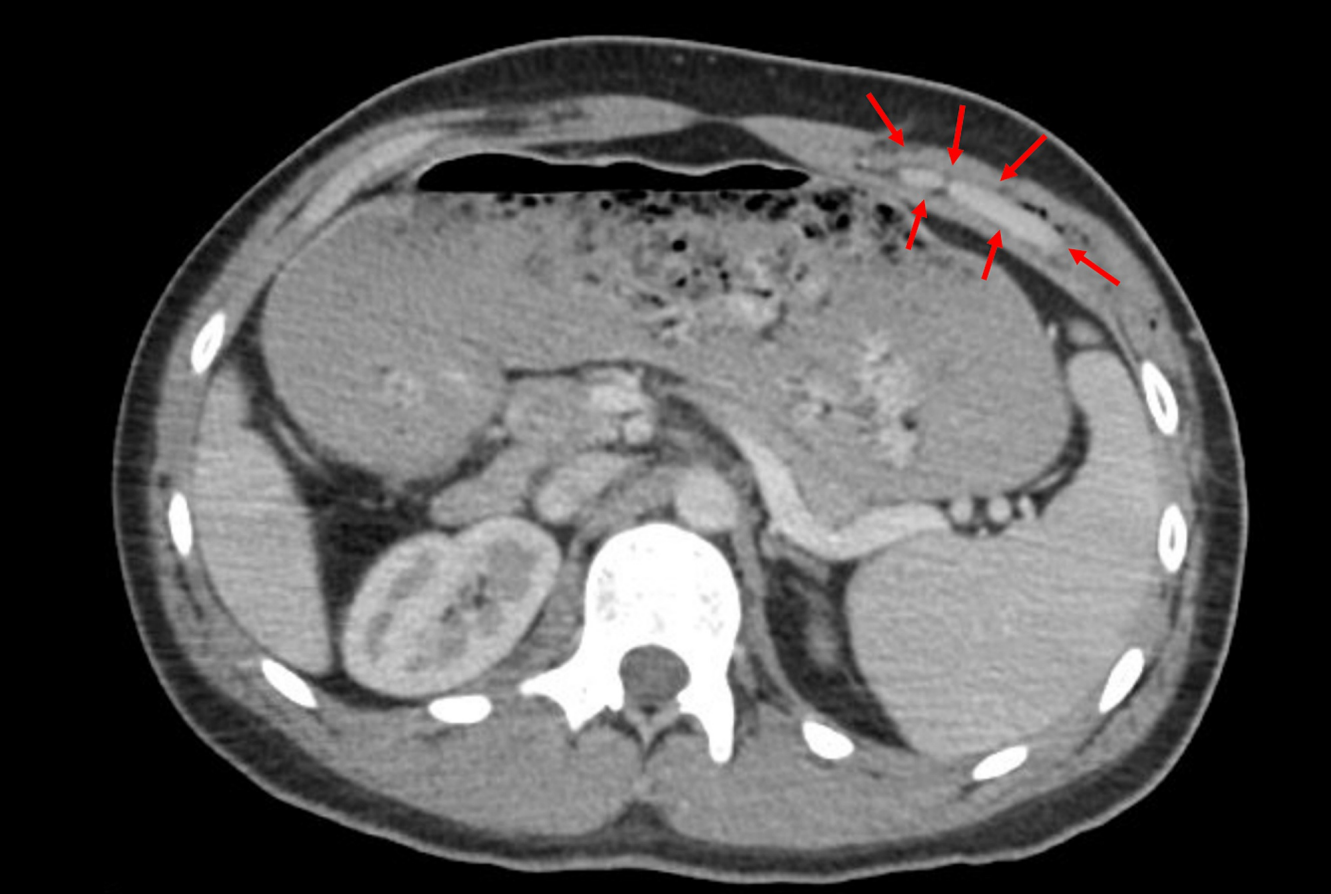
Initial ED CT with contrast revealed no evidence of a diaphragm or visceral injury. An upper left anterolateral abdominal wall hematoma is marked with red arrows, in axial view.

A 23-year-old male presented to his local rural ED in the middle of the night with a 2.5 cm stab wound to the left seventh intercostal space at the mid-axillary line. The patient reported getting into an altercation, leading to a nonaccidental injury. The stabbing happened within 30 minutes prior to his arrival. On initial physical examination, the lungs were clear to auscultation with symmetric expansion, there was no respiratory distress, and the abdomen was soft, nondistended, and nontender to palpation. Chest X-ray showed no radiographic abnormalities, and CT with contrast of the chest and abdomen demonstrated a left upper anterolateral abdominal wall hematoma without abdomino-pelvic visceral and diaphragmatic injury (Figure 1). His laboratory values were within normal limits. The patient’s wound was cleaned, irrigated, and closed with several sutures, and he was subsequently released from the ED. 

**Figure 2:**
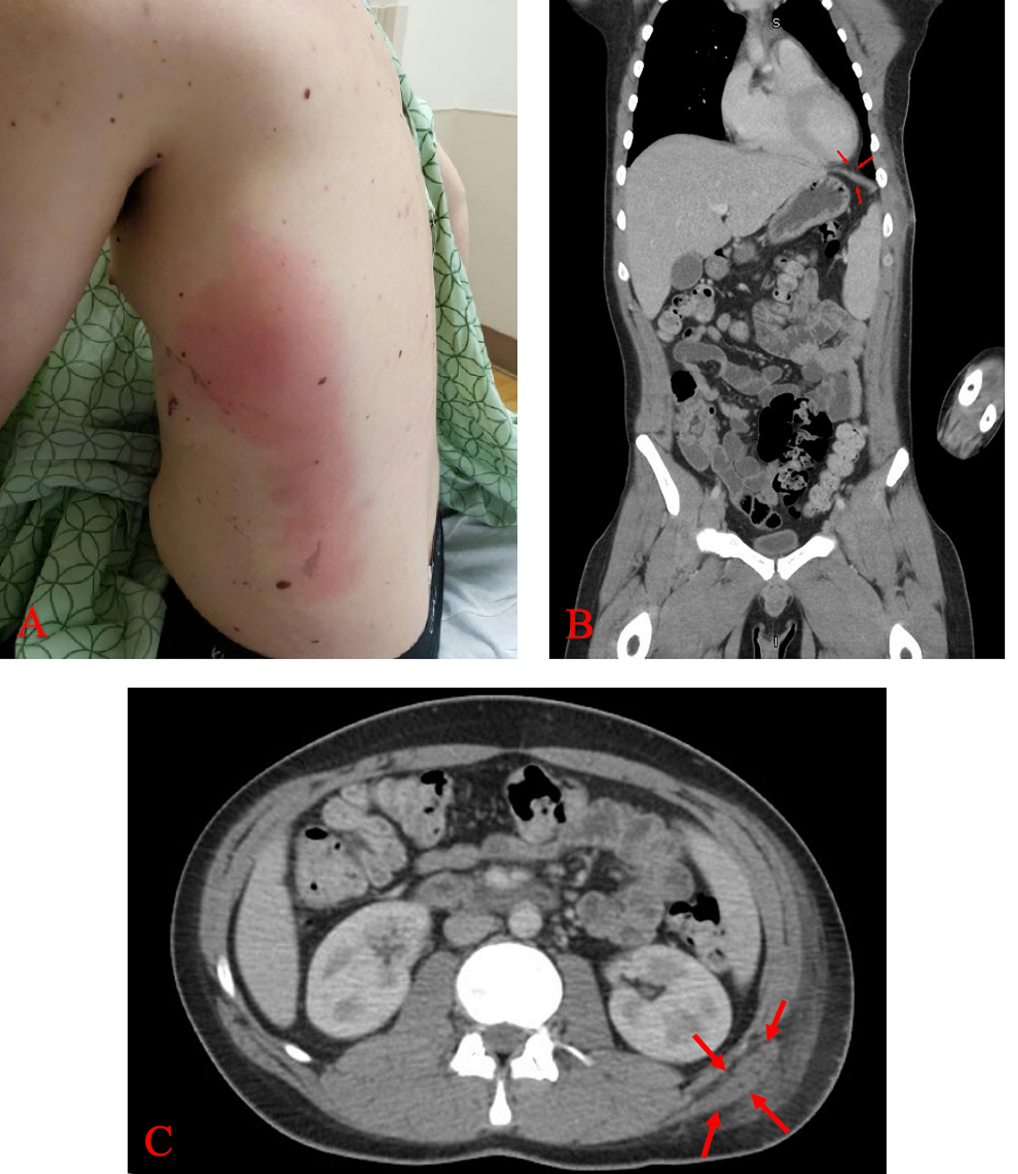
Repeat examination and CT with contrast five days after the initial stab wound. A: There is erythema overlying the area of the initial stab wound, left lateral posterior chest wall, and left flank. B: Fat stranding extending into the subcutaneous fat of the thorax near the left posterior flank is marked with red arrows, indicative of a traumatic diaphragmatic hernia. C: An abscess has formed around the left posterior thorax as indicated by the red arrows.

Some five days after the insult, he returned to the ED reporting fever, vomiting, left upper quadrant abdominal pain, and redness. Physical examination at this time showed local erythema of the wound itself, fluid density collection within intercostal muscles, and induration over left flank and left lateral posterior chest wall (Figure 2A). Repeat labs revealed leukocytosis (14.7 cells/L), elevated C-reactive peptide (293.3 mg/L), and elevated erythrocyte sedimentation rate (47 mm/h). Repeat CT with contrast illustrated significant enlargement of the left chest wall musculature, which was not seen initially. An abscess was formed, and there was fat stranding extending into the subcutaneous fat of the thorax near the left flank region (Figure 2B,C). 

He was admitted to the hospital for wound exploration. The patient underwent bedside removal of the sutures placed during his first visit, and copious amounts of pus drained from the wound immediately. Culture of the aspirate was later identified to be group A streptococcus. The wound was left open to drain, and ceftriaxone and metronidazole were initiated.

Initial surgery involved exploration of the stab wound and tracking of it medically towards the sternum and posteriorly towards the area of redness. There was avulsion of the intercostal muscles between the seventh and eighth ribs, and underlying tissue was palpated. 

**Figure 3:**
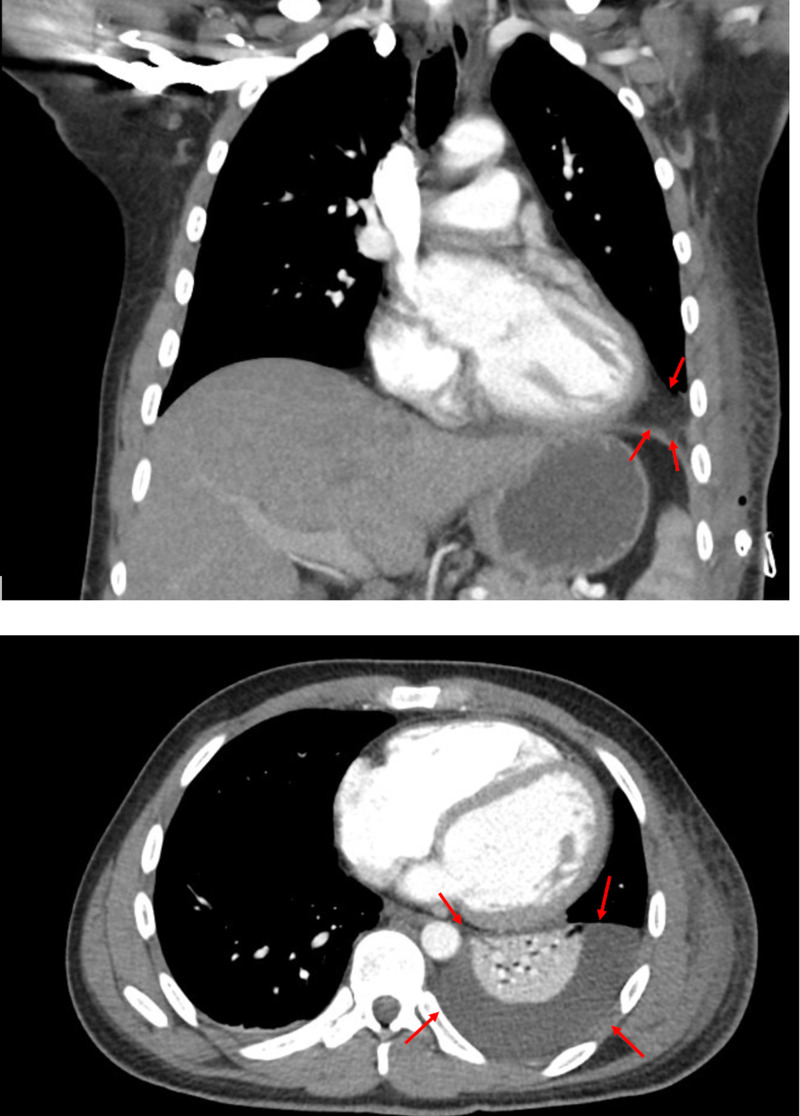
Repeat CT with contrast on day 3 of hospitalization. The initial insult evolved to a small consolidation and pleural effusion which are marked with red arrows in the coronal and axial views on CT scan.

Given the new CT and surgical findings, traumatic diaphragmatic hernia was suspected. Surgical repair was scheduled for the ensuing days. However, two days prior to operating, repeat CT showed atelectasis and moderate pleural effusion that was concerning for empyema (Figure 3). A chest tube was placed to mitigate symptoms prior to surgery. 

**Figure 4:**
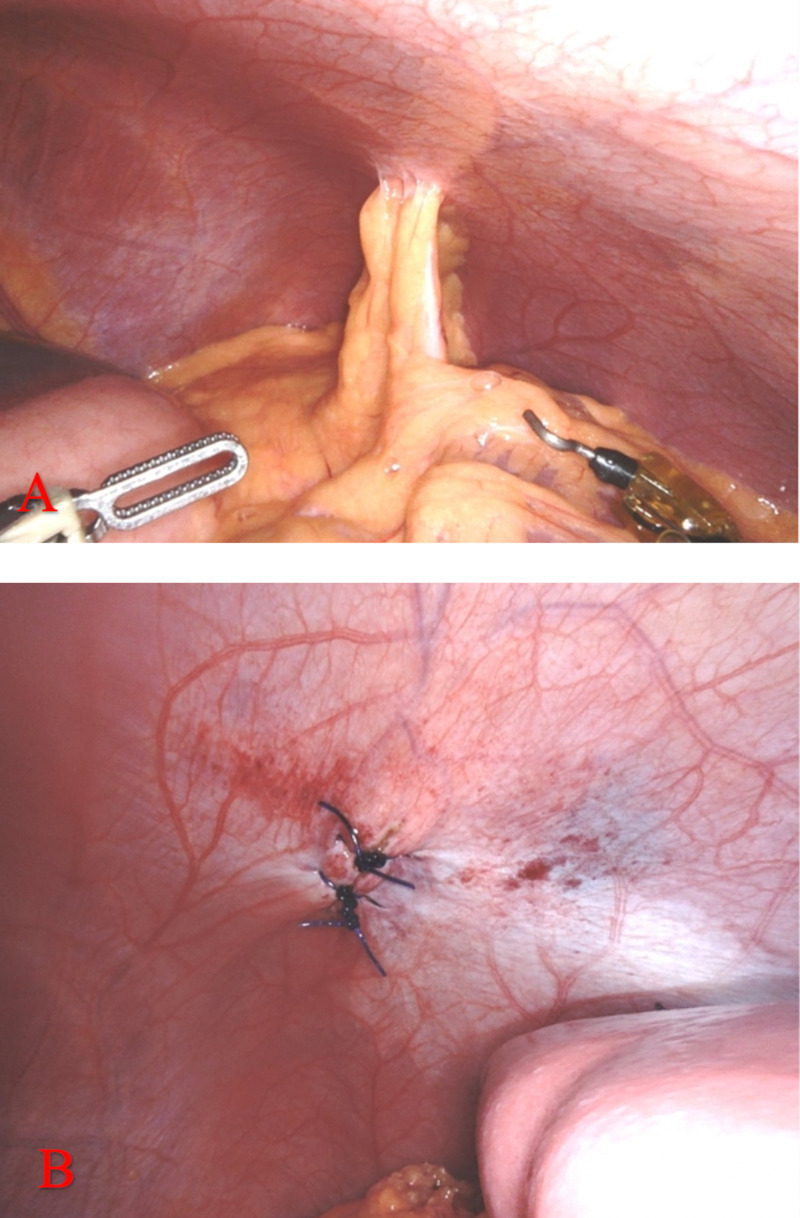
Repair of the injury took place on day 6 of hospitalization. A: Visualization of diaphragmatic hernia containing omental fat. Notably, there are no visceral organs that have entered through the defect. B: Correction of diaphragm defect with two simple interrupted polydioxanone sutures. There was no need for mesh as the edges of the defect could be approximated using sutures.

The patient underwent surgical repair of the suspected injury. A Visiport camera was placed in the supraumbilical region to access the peritoneal cavity under direct visualization. Additional trocars were placed left lower quadrant and right upper quadrant. Omentum was noted entering the left anterior 2 cm diaphragm defect, confirming the suspicions for diaphragm penetration (Figure 4A). The omentum was reduced into the abdominal cavity, and the defect was closed with two simple interrupted sutures using 0 polydiaxonone suture (Figure 4B).

There were no postoperative complications several days after the procedure, and he was discharged after a nine-day hospital stay. The patient was followed up in outpatient clinic a week after his operation. His wound was well-appearing and well-healing after cleaning and packing the wound on a regular basis. He had repeat labs and X-ray which demonstrated resolution of all previous findings. He was told to return for one last follow-up visit but did not return. 

## Discussion

Traumatic diaphragmatic hernias are proposed to occur secondary to a sudden increase in the pleuroperitoneal pressure gradient, and this occurs at weak points along embryological fusion points. The left diaphragm is generally considered weaker due to lack of protection by the liver [5, 11]. Our patient’s traumatic diaphragmatic hernia on the left side supports the findings in one retrospective study of 731 patients, where the left posterolateral region medial to the spleen was the most common site of injury [4].

It has been reported by Reber et al. that the rate of initially missed diaphragm damage might be as high as 66%. The risk of missing diaphragm injury is present even in patients who underwent laparotomy during initial examination of penetrating thoracoabdominal injury [12]. Miller et al. reported in their case series that 43% of patients (40/93) with penetrating injuries of the diaphragm had radiographs interpreted as normal [13]. Another study corroborated these findings, demonstrating that conventional plain X-ray, ultrasonography, and CT scan were not useful in diagnosing an acute diaphragmatic injury despite proving useful for injuries where visceral organ herniation was already present [14]. However, the data from the study by Lu et al. indicated benefits in using helical or multi-slice CT, as they allow pinpointing of more subtle signs of diaphragmatic injury. Their advantage lies in a dramatic reduction in beam-hardening artifacts, motion artifacts, and improved spatial resolution, all of which confer higher sensitivity, specificity, and accuracy compared to traditional acute radiographic methods. However, it has been shown that smaller, rural, and critical access hospitals have lower CT imaging availability and less access to higher-resolution CT scanners. Also, the availability of helical or multi-slice CT for ED patients remains unknown due to high cost, delivery of higher doses of radiation, and more time required to analyze data [8,14]. 

Once diagnosed, operative management of traumatic diaphragmatic hernia is pivotal to reduce the risk of subsequent complications. There are numerous methods to repair traumatic diaphragmatic hernia including by laparotomy, thoracotomy, laparoscopy, or thoracoscopy. Minimally invasive management is preferred for diaphragmatic injury when there is no concern for concurrent abdominal injuries [15]. Furthermore, minor amounts of diaphragmatic tissue destruction should be sufficient to approximate the initial laceration for primary repair without the need for mesh material, assuming there is no need for debridement. On the other hand, a larger amount diaphragmatic tissue loss or evolution of the primary defect are indications for usage of a nonabsorbable meshwork [15-16]. In case of contamination, copious irrigation should be used followed by placement of an autologous tissue flap or bioprosthesis [16]. 

## Conclusions

Despite advances in radiographic tools, physicians must not acquire a false sense of security in the setting of negative imaging or physical exam for penetrating trauma. Penetrating trauma in the area between the umbilicus and nipples anteriorly and posterior should be assumed to have a traumatic diaphragmatic injury until there is evidence to support the contrary. Delayed time to diagnosis is associated with significant rates mortality at 25%, as reported by the NTDB. Patients identified to have a diaphragmatic injury should undergo surgical repair of the injury which can be done openly, laparoscopically, or robotically. Future research should aim to investigate the use of helical CT in the ED and track the incidence of diaphragmatic injury in penetrating trauma.
